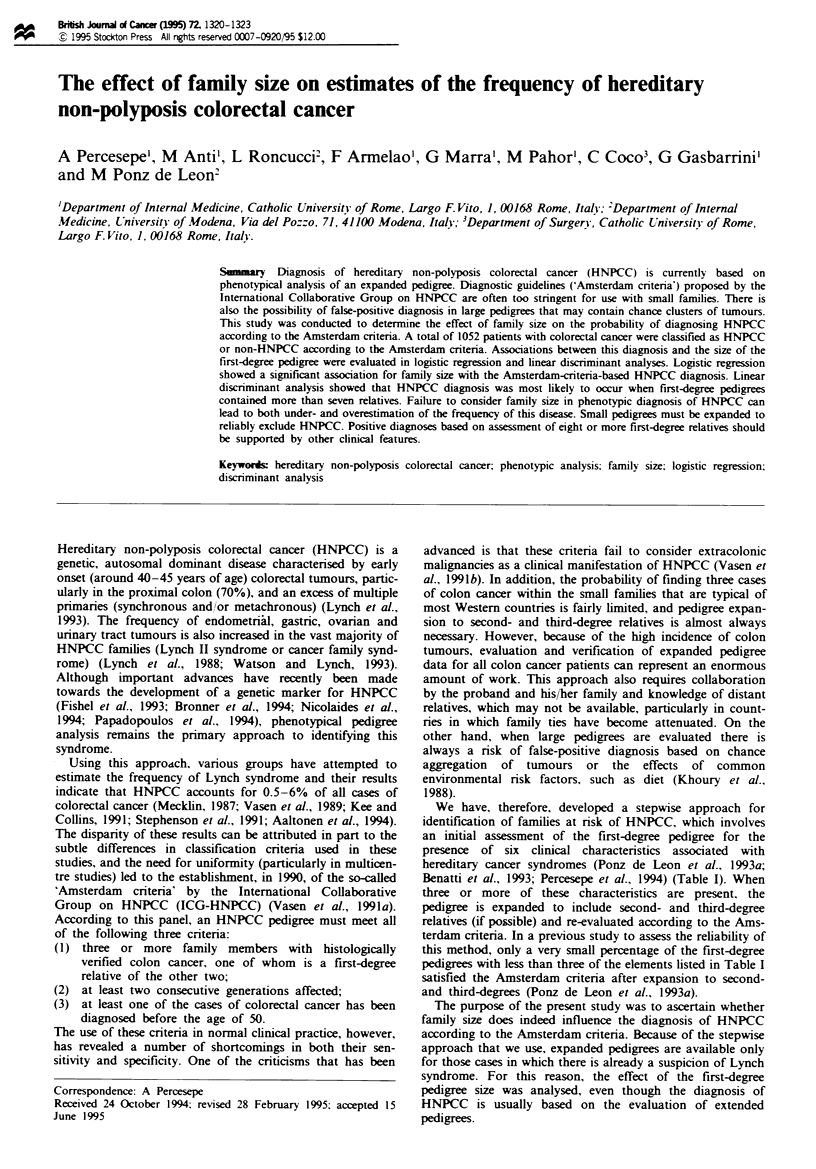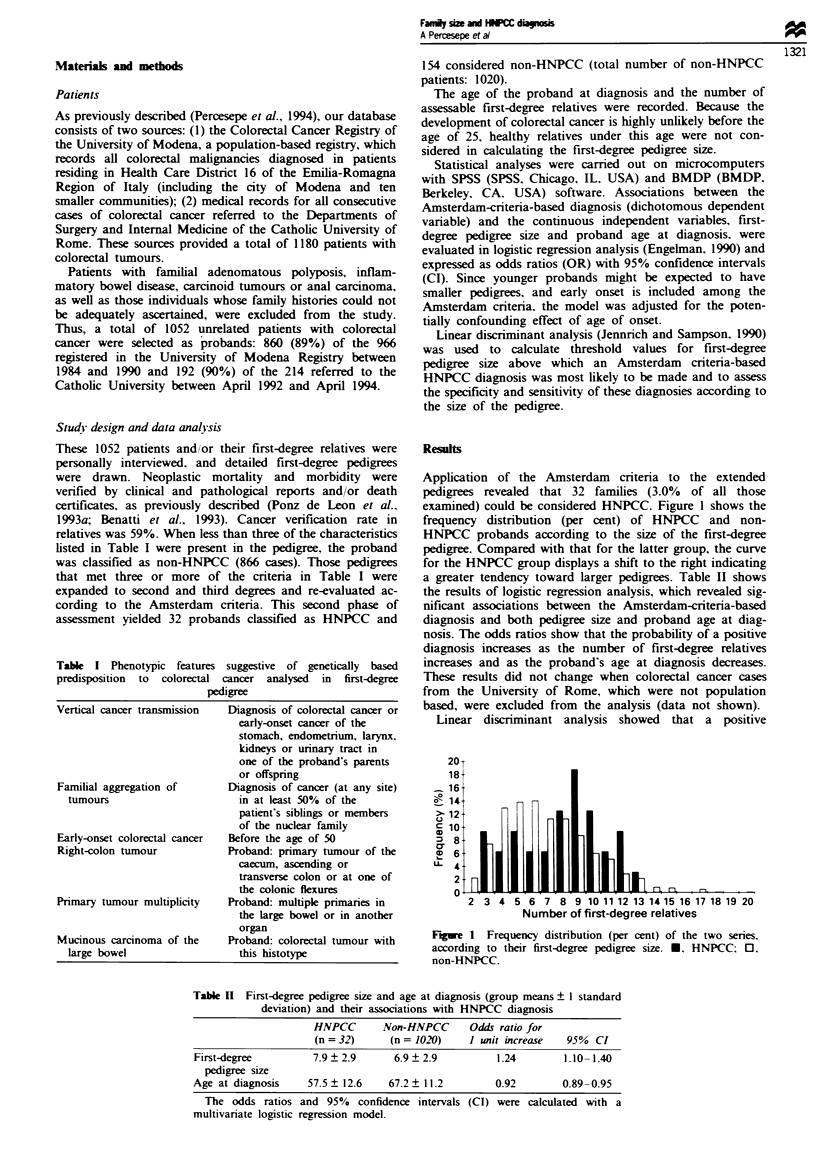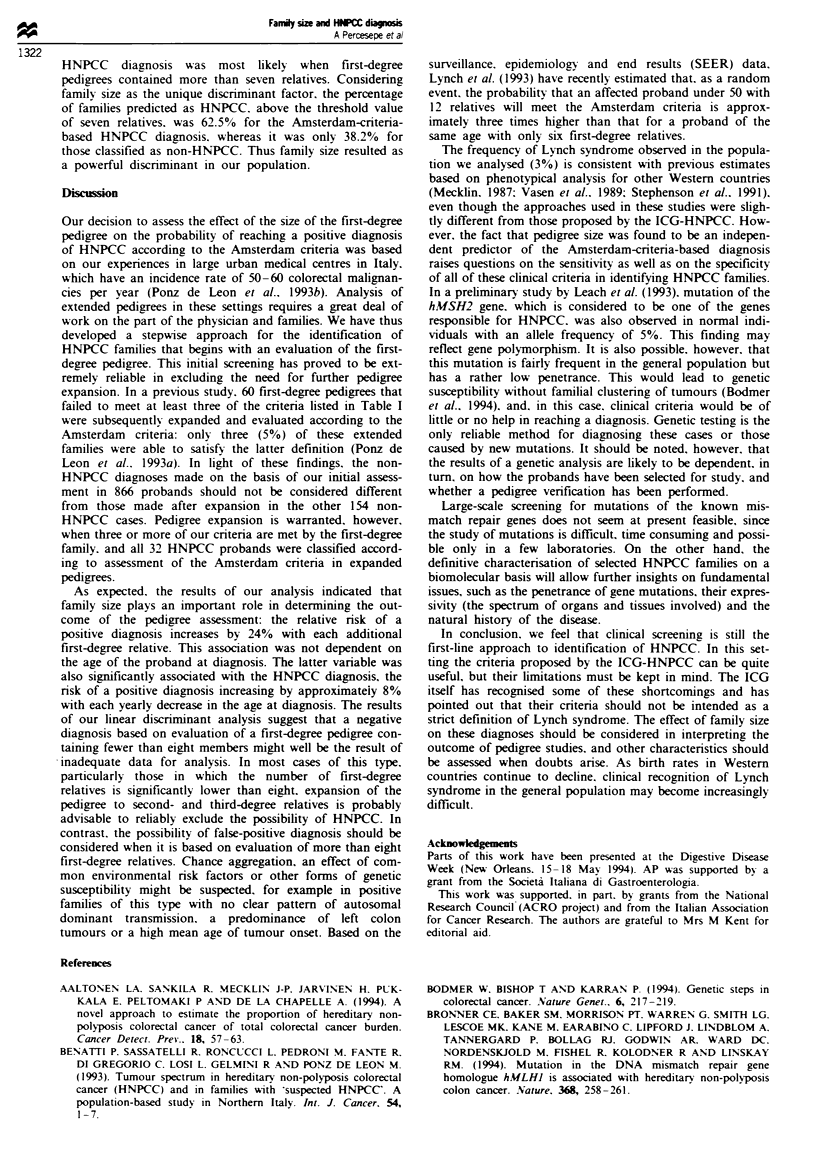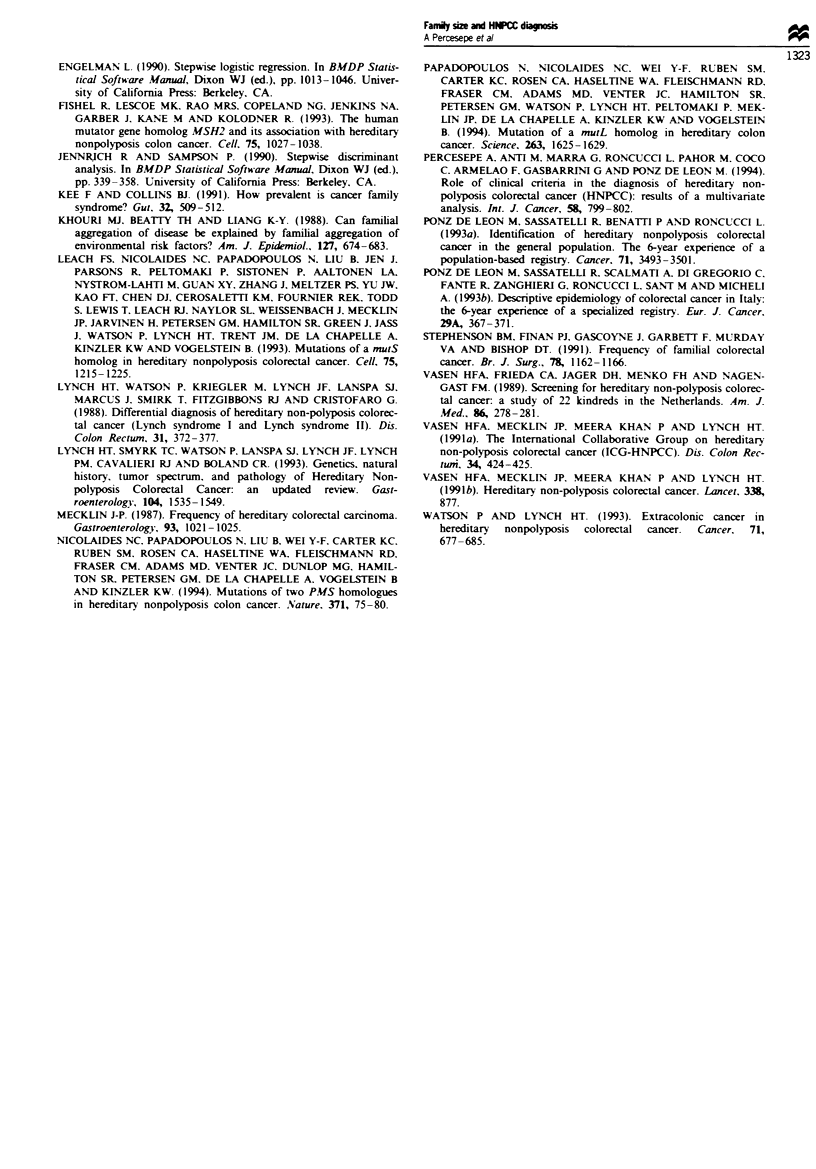# The effect of family size on estimates of the frequency of hereditary non-polyposis colorectal cancer.

**DOI:** 10.1038/bjc.1995.508

**Published:** 1995-11

**Authors:** A. Percesepe, M. Anti, L. Roncucci, F. Armelao, G. Marra, M. Pahor, C. Coco, G. Gasbarrini, M. Ponz de Leon

**Affiliations:** Department of Internal Medicine, Catholic University of Rome, Italy.

## Abstract

Diagnosis of hereditary non-polyposis colorectal cancer (HNPCC) is currently based on phenotypical analysis of an expanded pedigree. Diagnostic guidelines ('Amsterdam criteria') proposed by the International Collaborative Group on HNPCC are often too stringent for use with small families. There is also the possibility of false-positive diagnosis in large pedigrees that may contain chance clusters of tumours. This study was conducted to determine the effect of family size on the probability of diagnosing HNPCC according to the Amsterdam criteria. A total of 1052 patients with colorectal cancer were classified as HNPCC or non-HNPCC according to the Amsterdam criteria. Associations between this diagnosis and the size of the first-degree pedigree were evaluated in logistic regression and linear discriminant analyses. Logistic regression showed a significant association for family size with the Amsterdam-criteria-based HNPCC diagnosis. Linear discriminant analysis showed that HNPCC diagnosis was most likely to occur when first-degree pedigrees contained more than seven relatives. Failure to consider family size in phenotypic diagnosis of HNPCC can lead to both under- and overestimation of the frequency of this disease. Small pedigrees must be expanded to reliably exclude HNPCC. Positive diagnoses based on assessment of eight or more first-degree relatives should be supported by other clinical features.


British Journal of Cancer (1995) 72, 1320-1323
© 1995 Stockton Press All rights reserved 0007-0920/95 $12.00

### The effect of family size on estimates of the frequency of hereditary non-polyposis colorectal cancer

A Percesepe[1], M Anti[1], L Roncucci[2], F Armelao[1], G Marra[1], M Pahor[1], C Coco[3], G Gasbarrini[1] and M Ponz de Leon[2]

[1]*Department of Internal Medicine, Catholic University of Rome, Largo F.Vito, 1, 00168 Rome, Italy;* [2]*Department of Internal Medicine, University of Modena, Via del Pozzo, 71, 41100 Modena, Italy;* [3]*Department of Surgery, Catholic University of Rome, Largo F.Vito, 1, 00168 Rome, Italy.*

**Summary** Diagnosis of hereditary non-polyposis colorectal cancer (HNPCC) is currently based on phenotypical analysis of an expanded pedigree. Diagnostic guidelines ('Amsterdam criteria') proposed by the International Collaborative Group on HNPCC are often too stringent for use with small families. There is also the possibility of false-positive diagnosis in large pedigrees that may contain chance clusters of tumours. This study was conducted to determine the effect of family size on the probability of diagnosing HNPCC according to the Amsterdam criteria. A total of 1052 patients with colorectal cancer were classified as HNPCC or non-HNPCC according to the Amsterdam criteria. Associations between this diagnosis and the size of the first-degree pedigree were evaluated in logistic regression and linear discriminant analyses. Logistic regression showed a significant association for family size with the Amsterdam-criteria-based HNPCC diagnosis. Linear discriminant analysis showed that HNPCC diagnosis was most likely to occur when first-degree pedigrees contained more than seven relatives. Failure to consider family size in phenotypic diagnosis of HNPCC can lead to both under- and overestimation of the frequency of this disease. Small pedigrees must be expanded to reliably exclude HNPCC. Positive diagnoses based on assessment of eight or more first-degree relatives should be supported by other clinical features.

**Keywords:** hereditary non-polyposis colorectal cancer; phenotypic analysis; family size; logistic regression; discriminant analysis

Hereditary non-polyposis colorectal cancer (HNPCC) is a genetic, autosomal dominant disease characterised by early onset (around 40-45 years of age) colorectal tumours, particularly in the proximal colon (70%), and an excess of multiple primaries (synchronous and/or metachronous) (Lynch *et al.*, 1993). The frequency of endometrial, gastric, ovarian and urinary tract tumours is also increased in the vast majority of HNPCC families (Lynch II syndrome or cancer family syndrome) (Lynch *et al.*, 1988; Watson and Lynch, 1993). Although important advances have recently been made towards the development of a genetic marker for HNPCC (Fishel *et al.*, 1993; Bronner *et al.*, 1994; Nicolaides *et al.*, 1994; Papadopoulos *et al.*, 1994), phenotypical pedigree analysis remains the primary approach to identifying this syndrome.

Using this approach, various groups have attempted to estimate the frequency of Lynch syndrome and their results indicate that HNPCC accounts for 0.5-6% of all cases of colorectal cancer (Mecklin, 1987; Vasen *et al.*, 1989; Kee and Collins, 1991; Stephenson *et al.*, 1991; Aaltonen *et al.*, 1994). The disparity of these results can be attributed in part to the subtle differences in classification criteria used in these studies, and the need for uniformity (particularly in multicentre studies) led to the establishment, in 1990, of the so-called 'Amsterdam criteria' by the International Collaborative Group on HNPCC (ICG-HNPCC) (Vasen *et al.*, 1991*a*). According to this panel, an HNPCC pedigree must meet all of the following three criteria:

(1) three or more family members with histologically verified colon cancer, one of whom is a first-degree relative of the other two;
(2) at least two consecutive generations affected;
(3) at least one of the cases of colorectal cancer has been diagnosed before the age of 50.

The use of these criteria in normal clinical practice, however, has revealed a number of shortcomings in both their sensitivity and specificity. One of the criticisms that has been advanced is that these criteria fail to consider extracolonic malignancies as a clinical manifestation of HNPCC (Vasen *et al.*, 1991*b*). In addition, the probability of finding three cases of colon cancer within the small families that are typical of most Western countries is fairly limited, and pedigree expansion to second- and third-degree relatives is almost always necessary. However, because of the high incidence of colon tumours, evaluation and verification of expanded pedigree data for all colon cancer patients can represent an enormous amount of work. This approach also requires collaboration by the proband and his/her family and knowledge of distant relatives, which may not be available, particularly in countries in which family ties have become attenuated. On the other hand, when large pedigrees are evaluated there is always a risk of false-positive diagnosis based on chance aggregation of tumours or the effects of common environmental risk factors, such as diet (Khoury *et al.*, 1988).

We have, therefore, developed a stepwise approach for identification of families at risk of HNPCC, which involves an initial assessment of the first-degree pedigree for the presence of six clinical characteristics associated with hereditary cancer syndromes (Ponz de Leon *et al.*, 1993*a*; Benatti *et al.*, 1993; Percesepe *et al.*, 1994) (Table I). When three or more of these characteristics are present, the pedigree is expanded to include second- and third-degree relatives (if possible) and re-evaluated according to the Amsterdam criteria. In a previous study to assess the reliability of this method, only a very small percentage of the first-degree pedigrees with less than three of the elements listed in Table I satisfied the Amsterdam criteria after expansion to second- and third-degrees (Ponz de Leon *et al.*, 1993*a*).

The purpose of the present study was to ascertain whether family size does indeed influence the diagnosis of HNPCC according to the Amsterdam criteria. Because of the stepwise approach that we use, expanded pedigrees are available only for those cases in which there is already a suspicion of Lynch syndrome. For this reason, the effect of the first-degree pedigree size was analysed, even though the diagnosis of HNPCC is usually based on the evaluation of extended pedigrees.

Correspondence: A Percesepe
Received 24 October 1994; revised 28 February 1995; accepted 15 June 1995

### Materials and methods

### *Patients*

As previously described (Percesepe *et al*., 1994), our database consists of two sources: (1) the Colorectal Cancer Registry of the University of Modena, a population-based registry, which records all colorectal malignancies diagnosed in patients residing in Health Care District 16 of the Emilia-Romagna Region of Italy (including the city of Modena and ten smaller communities); (2) medical records for all consecutive cases of colorectal cancer referred to the Departments of Surgery and Internal Medicine of the Catholic University of Rome. These sources provided a total of 1180 patients with colorectal tumours.

Patients with familial adenomatous polyposis, inflammatory bowel disease, carcinoid tumours or anal carcinoma, as well as those individuals whose family histories could not be adequately ascertained, were excluded from the study. Thus, a total of 1052 unrelated patients with colorectal cancer were selected as probands: 860 (89%) of the 966 registered in the University of Modena Registry between 1984 and 1990 and 192 (90%) of the 214 referred to the Catholic University between April 1992 and April 1994.

### *Study design and data analysis*

These 1052 patients and/or their first-degree relatives were personally interviewed, and detailed first-degree pedigrees were drawn. Neoplastic mortality and morbidity were verified by clinical and pathological reports and/or death certificates, as previously described (Ponz de Leon *et al*., 1993*a*; Benatti *et al*., 1993). Cancer verification rate in relatives was 59%. When less than three of the characteristics listed in Table I were present in the pedigree, the proband was classified as non-HNPCC (866 cases). Those pedigrees that met three or more of the criteria in Table I were expanded to second and third degrees and re-evaluated according to the Amsterdam criteria. This second phase of assessment yielded 32 probands classified as HNPCC and 154 considered non-HNPCC (total number of non-HNPCC patients: 1020).

The age of the proband at diagnosis and the number of assessable first-degree relatives were recorded. Because the development of colorectal cancer is highly unlikely before the age of 25, healthy relatives under this age were not considered in calculating the first-degree pedigree size.

Statistical analyses were carried out on microcomputers with SPSS (SPSS, Chicago, IL, USA) and BMDP (BMDP, Berkeley, CA, USA) software. Associations between the Amsterdam-criteria-based diagnosis (dichotomous dependent variable) and the continuous independent variables, first-degree pedigree size and proband age at diagnosis, were evaluated in logistic regression analysis (Engelman, 1990) and expressed as odds ratios (OR) with 95% confidence intervals (CI). Since younger probands might be expected to have smaller pedigrees, and early onset is included among the Amsterdam criteria, the model was adjusted for the potentially confounding effect of age of onset.

Linear discriminant analysis (Jennrich and Sampson, 1990) was used to calculate threshold values for first-degree pedigree size above which an Amsterdam criteria-based HNPCC diagnosis was most likely to be made and to assess the specificity and sensitivity of these diagnosies according to the size of the pedigree.

**Table I** Phenotypic features suggestive of genetically based predisposition to colorectal cancer analysed in first-degree pedigree

| | |
|---|---|
| Vertical cancer transmission | Diagnosis of colorectal cancer or early-onset cancer of the stomach, endometrium, larynx, kidneys or urinary tract in one of the proband's parents or offspring |
| Familial aggregation of tumours | Diagnosis of cancer (at any site) in at least 50% of the patient's siblings or members of the nuclear family |
| Early-onset colorectal cancer | Before the age of 50 |
| Right-colon tumour | Proband: primary tumour of the caecum, ascending or transverse colon or at one of the colonic flexures |
| Primary tumour multiplicity | Proband: multiple primaries in the large bowel or in another organ |
| Mucinous carcinoma of the large bowel | Proband: colorectal tumour with this histotype |

### Results

Application of the Amsterdam criteria to the extended pedigrees revealed that 32 families (3.0% of all those examined) could be considered HNPCC. Figure 1 shows the frequency distribution (per cent) of HNPCC and non-HNPCC probands according to the size of the first-degree pedigree. Compared with that for the latter group, the curve for the HNPCC group displays a shift to the right indicating a greater tendency toward larger pedigrees. Table II shows the results of logistic regression analysis, which revealed significant associations between the Amsterdam-criteria-based diagnosis and both pedigree size and proband age at diagnosis. The odds ratios show that the probability of a positive diagnosis increases as the number of first-degree relatives increases and as the proband's age at diagnosis decreases. These results did not change when colorectal cancer cases from the University of Rome, which were not population based, were excluded from the analysis (data not shown).

Linear discriminant analysis showed that a positive

**Figure 1** Frequency distribution (per cent) of the two series, according to their first-degree pedigree size. ■, HNPCC; □, non-HNPCC.

**Table II** First-degree pedigree size and age at diagnosis (group means ± 1 standard deviation) and their associations with HNPCC diagnosis

| | *HNPCC* (n = *32*) | *Non-HNPCC* (n = *1020*) | *Odds ratio for 1 unit increase* | *95% CI* |
|---|---|---|---|---|
| First-degree pedigree size | 7.9 ± 2.9 | 6.9 ± 2.9 | 1.24 | 1.10–1.40 |
| Age at diagnosis | 57.5 ± 12.6 | 67.2 ± 11.2 | 0.92 | 0.89–0.95 |

The odds ratios and 95% confidence intervals (CI) were calculated with a multivariate logistic regression model.

HNPCC diagnosis was most likely when first-degree pedigrees contained more than seven relatives. Considering family size as the unique discriminant factor, the percentage of families predicted as HNPCC, above the threshold value of seven relatives, was 62.5% for the Amsterdam-criteria-based HNPCC diagnosis, whereas it was only 38.2% for those classified as non-HNPCC. Thus family size resulted as a powerful discriminant in our population.

### Discussion

Our decision to assess the effect of the size of the first-degree pedigree on the probability of reaching a positive diagnosis of HNPCC according to the Amsterdam criteria was based on our experiences in large urban medical centres in Italy, which have an incidence rate of 50–60 colorectal malignancies per year (Ponz de Leon *et al*., 1993*b*). Analysis of extended pedigrees in these settings requires a great deal of work on the part of the physician and families. We have thus developed a stepwise approach for the identification of HNPCC families that begins with an evaluation of the first-degree pedigree. This initial screening has proved to be extremely reliable in excluding the need for further pedigree expansion. In a previous study, 60 first-degree pedigrees that failed to meet at least three of the criteria listed in Table I were subsequently expanded and evaluated according to the Amsterdam criteria: only three (5%) of these extended families were able to satisfy the latter definition (Ponz de Leon *et al*., 1993*a*). In light of these findings, the non-HNPCC diagnoses made on the basis of our initial assessment in 866 probands should not be considered different from those made after expansion in the other 154 non-HNPCC cases. Pedigree expansion is warranted, however, when three or more of our criteria are met by the first-degree family, and all 32 HNPCC probands were classified according to assessment of the Amsterdam criteria in expanded pedigrees.

As expected, the results of our analysis indicated that family size plays an important role in determining the outcome of the pedigree assessment: the relative risk of a positive diagnosis increases by 24% with each additional first-degree relative. This association was not dependent on the age of the proband at diagnosis. The latter variable was also significantly associated with the HNPCC diagnosis, the risk of a positive diagnosis increasing by approximately 8% with each yearly decrease in the age at diagnosis. The results of our linear discriminant analysis suggest that a negative diagnosis based on evaluation of a first-degree pedigree containing fewer than eight members might well be the result of inadequate data for analysis. In most cases of this type, particularly those in which the number of first-degree relatives is significantly lower than eight, expansion of the pedigree to second- and third-degree relatives is probably advisable to reliably exclude the possibility of HNPCC. In contrast, the possibility of false-positive diagnosis should be considered when it is based on evaluation of more than eight first-degree relatives. Chance aggregation, an effect of common environmental risk factors or other forms of genetic susceptibility might be suspected, for example in positive families of this type with no clear pattern of autosomal dominant transmission, a predominance of left colon tumours or a high mean age of tumour onset. Based on the surveillance, epidemiology and end results (SEER) data, Lynch *et al*. (1993) have recently estimated that, as a random event, the probability that an affected proband under 50 with 12 relatives will meet the Amsterdam criteria is approximately three times higher than that for a proband of the same age with only six first-degree relatives.

The frequency of Lynch syndrome observed in the population we analysed (3%) is consistent with previous estimates based on phenotypical analysis for other Western countries (Mecklin, 1987; Vasen *et al*., 1989; Stephenson *et al*., 1991), even though the approaches used in these studies were slightly different from those proposed by the ICG-HNPCC. However, the fact that pedigree size was found to be an independent predictor of the Amsterdam-criteria-based diagnosis raises questions on the sensitivity as well as on the specificity of all of these clinical criteria in identifying HNPCC families. In a preliminary study by Leach *et al*. (1993), mutation of the *hMSH2* gene, which is considered to be one of the genes responsible for HNPCC, was also observed in normal individuals with an allele frequency of 5%. This finding may reflect gene polymorphism. It is also possible, however, that this mutation is fairly frequent in the general population but has a rather low penetrance. This would lead to genetic susceptibility without familial clustering of tumours (Bodmer *et al*., 1994), and, in this case, clinical criteria would be of little or no help in reaching a diagnosis. Genetic testing is the only reliable method for diagnosing these cases or those caused by new mutations. It should be noted, however, that the results of a genetic analysis are likely to be dependent, in turn, on how the probands have been selected for study, and whether a pedigree verification has been performed.

Large-scale screening for mutations of the known mismatch repair genes does not seem at present feasible, since the study of mutations is difficult, time consuming and possible only in a few laboratories. On the other hand, the definitive characterisation of selected HNPCC families on a biomolecular basis will allow further insights on fundamental issues, such as the penetrance of gene mutations, their expressivity (the spectrum of organs and tissues involved) and the natural history of the disease.

In conclusion, we feel that clinical screening is still the first-line approach to identification of HNPCC. In this setting the criteria proposed by the ICG-HNPCC can be quite useful, but their limitations must be kept in mind. The ICG itself has recognised some of these shortcomings and has pointed out that their criteria should not be intended as a strict definition of Lynch syndrome. The effect of family size on these diagnoses should be considered in interpreting the outcome of pedigree studies, and other characteristics should be assessed when doubts arise. As birth rates in Western countries continue to decline, clinical recognition of Lynch syndrome in the general population may become increasingly difficult.

### Acknowledgements

Parts of this work have been presented at the Digestive Disease Week (New Orleans, 15–18 May 1994). AP was supported by a grant from the Società Italiana di Gastroenterologia.

This work was supported, in part, by grants from the National Research Council (ACRO project) and from the Italian Association for Cancer Research. The authors are grateful to Mrs M Kent for editorial aid.

### References

AALTONEN LA, SANKILA R, MECKLIN J-P, JARVINEN H, PUKKALA E, PELTOMAKI P AND DE LA CHAPELLE A. (1994). A novel approach to estimate the proportion of hereditary non-polyposis colorectal cancer of total colorectal cancer burden. *Cancer Detect. Prev.*, **18**, 57–63.

BENATTI P, SASSATELLI R, RONCUCCI L, PEDRONI M, FANTE R, DI GREGORIO C, LOSI L, GELMINI R AND PONZ DE LEON M. (1993). Tumour spectrum in hereditary non-polyposis colorectal cancer (HNPCC) and in families with 'suspected HNPCC'. A population-based study in Northern Italy. *Int. J. Cancer*, **54**, 1–7.

BODMER W, BISHOP T AND KARRAN P. (1994). Genetic steps in colorectal cancer. *Nature Genet.*, **6**, 217–219.

BRONNER CE, BAKER SM, MORRISON PT, WARREN G, SMITH LG, LESCOE MK, KANE M, EARABINO C, LIPFORD J, LINDBLOM A, TANNERGARD P, BOLLAG RJ, GODWIN AR, WARD DC, NORDENSKJOLD M, FISHEL R, KOLODNER R AND LINSKAY RM. (1994). Mutation in the DNA mismatch repair gene homologue *hMLH1* is associated with hereditary non-polyposis colon cancer. *Nature*, **368**, 258–261.

ENGELMAN L. (1990). Stepwise logistic regression. In *BMDP Statistical Software Manual*, Dixon WJ (ed.), pp. 1013–1046. University of California Press: Berkeley, CA.

FISHEL R, LESCOE MK, RAO MRS, COPELAND NG, JENKINS NA, GARBER J, KANE M AND KOLODNER R. (1993). The human mutator gene homolog *MSH2* and its association with hereditary nonpolyposis colon cancer. *Cell*, **75**, 1027–1038.

JENNRICH R AND SAMPSON P. (1990). Stepwise discriminant analysis. In *BMDP Statistical Software Manual*, Dixon WJ (ed.), pp. 339–358. University of California Press: Berkeley, CA.

KEE F AND COLLINS BJ. (1991). How prevalent is cancer family syndrome? *Gut*, **32**, 509–512.

KHOURI MJ, BEATTY TH AND LIANG K-Y. (1988). Can familial aggregation of disease be explained by familial aggregation of environmental risk factors? *Am. J. Epidemiol.*, **127**, 674–683.

LEACH FS, NICOLAIDES NC, PAPADOPOULOS N, LIU B, JEN J, PARSONS R, PELTOMAKI P, SISTONEN P, AALTONEN LA, NYSTROM-LAHTI M, GUAN XY, ZHANG J, MELTZER PS, YU JW, KAO FT, CHEN DJ, CEROSALETTI KM, FOURNIER REK, TODD S, LEWIS T, LEACH RJ, NAYLOR SL, WEISSENBACH J, MECKLIN JP, JARVINEN H, PETERSEN GM, HAMILTON SR, GREEN J, JASS J, WATSON P, LYNCH HT, TRENT JM, DE LA CHAPELLE A, KINZLER KW AND VOGELSTEIN B. (1993). Mutations of a *mutS* homolog in hereditary nonpolyposis colorectal cancer. *Cell*, **75**, 1215–1225.

LYNCH HT, WATSON P, KRIEGLER M, LYNCH JF, LANSPA SJ, MARCUS J, SMIRK T, FITZGIBBONS RJ AND CRISTOFARO G. (1988). Differential diagnosis of hereditary non-polyposis colorectal cancer (Lynch syndrome I and Lynch syndrome II). *Dis. Colon Rectum*, **31**, 372–377.

LYNCH HT, SMYRK TC, WATSON P, LANSPA SJ, LYNCH JF, LYNCH PM, CAVALIERI RJ AND BOLAND CR. (1993). Genetics, natural history, tumor spectrum, and pathology of Hereditary Nonpolyposis Colorectal Cancer: an updated review. *Gastroenterology*, **104**, 1535–1549.

MECKLIN J-P. (1987). Frequency of hereditary colorectal carcinoma. *Gastroenterology*, **93**, 1021–1025.

NICOLAIDES NC, PAPADOPOULOS N, LIU B, WEI Y-F, CARTER KC, RUBEN SM, ROSEN CA, HASELTINE WA, FLEISCHMANN RD, FRASER CM, ADAMS MD, VENTER JC, DUNLOP MG, HAMILTON SR, PETERSEN GM, DE LA CHAPELLE A, VOGELSTEIN B AND KINZLER KW. (1994). Mutations of two *PMS* homologues in hereditary nonpolyposis colon cancer. *Nature*, **371**, 75–80.

PAPADOPOULOS N, NICOLAIDES NC, WEI Y-F, RUBEN SM, CARTER KC, ROSEN CA, HASELTINE WA, FLEISCHMANN RD, FRASER CM, ADAMS MD, VENTER JC, HAMILTON SR, PETERSEN GM, WATSON P, LYNCH HT, PELTOMAKI P, MEKLIN JP, DE LA CHAPELLE A, KINZLER KW AND VOGELSTEIN B. (1994). Mutation of a *mutL* homolog in hereditary colon cancer. *Science*, **263**, 1625–1629.

PERCESEPE A, ANTI M, MARRA G, RONCUCCI L, PAHOR M, COCO C, ARMELAO F, GASBARRINI G AND PONZ DE LEON M. (1994). Role of clinical criteria in the diagnosis of hereditary nonpolyposis colorectal cancer (HNPCC): results of a multivariate analysis. *Int. J. Cancer*, **58**, 799–802.

PONZ DE LEON M, SASSATELLI R, BENATTI P AND RONCUCCI L. (1993*a*). Identification of hereditary nonpolyposis colorectal cancer in the general population. The 6-year experience of a population-based registry. *Cancer*, **71**, 3493–3501.

PONZ DE LEON M, SASSATELLI R, SCALMATI A, DI GREGORIO C, FANTE R, ZANGHIERI G, RONCUCCI L, SANT M AND MICHELI A. (1993*b*). Descriptive epidemiology of colorectal cancer in Italy: the 6-year experience of a specialized registry. *Eur. J. Cancer*, **29A**, 367–371.

STEPHENSON BM, FINAN PJ, GASCOYNE J, GARBETT F, MURDAY VA AND BISHOP DT. (1991). Frequency of familial colorectal cancer. *Br. J. Surg.*, **78**, 1162–1166.

VASEN HFA, FRIEDA CA, JAGER DH, MENKO FH AND NAGENGAST FM. (1989). Screening for hereditary non-polyposis colorectal cancer: a study of 22 kindreds in the Netherlands. *Am. J. Med.*, **86**, 278–281.

VASEN HFA, MECKLIN JP, MEERA KHAN P AND LYNCH HT. (1991*a*). The International Collaborative Group on hereditary non-polyposis colorectal cancer (ICG-HNPCC). *Dis. Colon Rectum*, **34**, 424–425.

VASEN HFA, MECKLIN JP, MEERA KHAN P AND LYNCH HT. (1991*b*). Hereditary non-polyposis colorectal cancer. *Lancet*, **338**, 877.

WATSON P AND LYNCH HT. (1993). Extracolonic cancer in hereditary nonpolyposis colorectal cancer. *Cancer*, **71**, 677–685.